# Licorice‐Induced Pseudoaldosteronism Caused by Rikkunshito in an Extremely Low Birth Weight Infant

**DOI:** 10.1111/ped.70407

**Published:** 2026-04-15

**Authors:** Toshihiro Nakayama, Naofumi Hanyu, Shonosuke Nara, Takashi Yamazaki, Gaku Yamanaka

**Affiliations:** ^1^ Department of Pediatrics and Adolescent Medicine Tokyo Medical University Tokyo Japan

**Keywords:** extremely low birth weight infant, gastroesophageal reflux disease, hypokalemia, Kampo, metabolic side effects of drugs

## Abstract

We report an extremely low birth weight infant with gastroesophageal reflux disease who developed persistent hypokalemia and metabolic alkalosis after treatment with rikkunshito. These findings gradually resolved after discontinuation of rikkunshito and potassium supplementation, suggesting licorice‐induced pseudoaldosteronism.

Gastroesophageal reflux disease (GERD) is common in early infancy, largely due to immaturity of the lower esophageal sphincter and gastrointestinal motility. Especially in preterm infants, GERD can trigger laryngeal stimulation and lead to apnea or bradycardia, which may require clinical management. Management involves frequent feedings, continuous enteral infusion via nasogastric or nasojejunal tube, positional therapy, and pharmacologic acid suppression. In recent years, traditional Japanese medicine (Kampo), particularly the formula rikkunshito, has gained attention as a pharmacologic approach to GERD and is now gradually being applied to children [1]. We report an extremely low birth weight infant with GERD who developed persistent hypokalemia after receiving Kampo therapy with rikkunshito, suggesting licorice‐induced pseudoaldosteronism.

The patient was delivered by emergency cesarean section at 24 weeks and 0 days of gestation because of non‐reassuring fetal status, with a birth weight of 516 g. The Apgar scores were 1 at 1 min and 6 at 5 min. Surfactant therapy and mechanical ventilation were started after birth for respiratory distress syndrome. On Day 14 of life, GERD‐associated bradycardia occurred frequently, and continuous 24‐h enteral feeding via gastric tubes and nasojejunal tubes was attempted. However, since the symptoms persisted, TSUMURA Rikkunshito Extract Granules for Ethical Use (0.15 g/kg/day of extract granules, TJ‐43; Tsumura & Co., Tokyo, Japan) were initiated on Day 28 of life. After initiation of rikkunshito, both reflux episodes and the subsequent reflux‐associated bradycardic events decreased in frequency and severity, and the same dosage was maintained. Subsequently, on Day 58 of life, blood testing revealed hypokalemia (2.0 mmol/L). The fractional excretion of potassium was 22.1%, suggesting renal potassium loss. Liver and cholestatic function were within normal limits, and no abnormality was detected in blood pressure, urine output or electrocardiography. Neither potassium‐lowering drugs such as diuretics and corticosteroids, nor doxapram, had been administered around the time of onset. Based on these findings, we discontinued rikkunshito due to suspicion of licorice‐induced pseudoaldosteronism, initiating oral potassium therapy (2 mEq/kg/day). Although the hypokalemia persisted for over a month, serum potassium levels gradually normalized, allowing discontinuation of oral potassium therapy by Day 100 of life, with no recurrence thereafter. Following the onset of hypokalemia, a marked increase in serum bicarbonate was observed. PaCO_2_ was mildly elevated above the institutional reference range, consistent with respiratory compensation maintaining normal blood pH. These abnormalities normalized in parallel with the correction of serum potassium (Figure 1).

**FIGURE 1:**
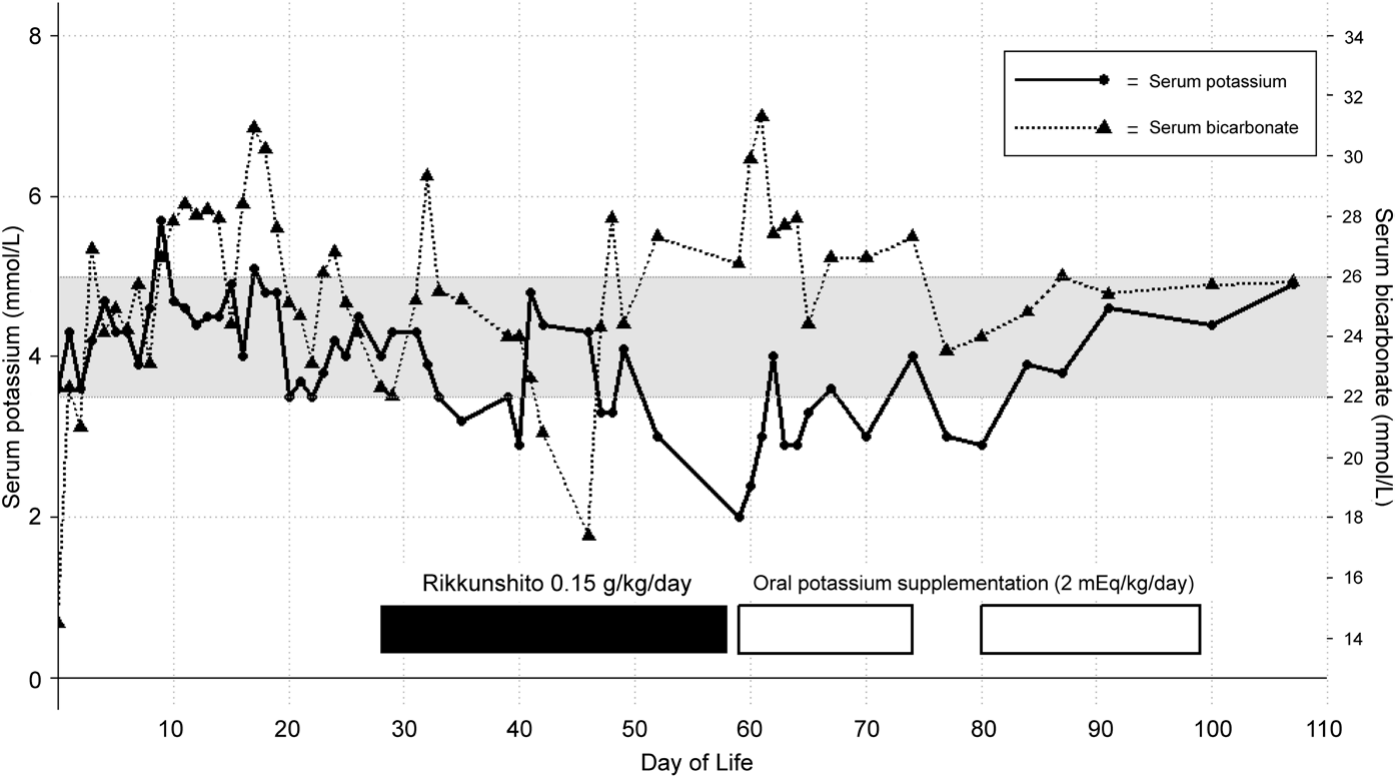
Trend in serum potassium (mmol/L) and bicarbonate (mmol/L) levels. The gray bands show normal ranges.

Rikkunshito is composed of eight crude drugs: *Atractylodis lanceae rhizoma*, *Ginseng radix*, *Pinelliae tuber*, *Poria sclerotium*, *Zizyphi fructus*, *Citri unshiu pericarpium*, *Glycyrrhizae radix* (licorice), and *Zingiberis rhizoma* (names according to the English version of the Japanese Pharmacopeia, 18th Edition). Active constituents such as isoliquiritigenin, hesperidin and atractylodin stimulate ghrelin secretion from gastric endocrine cells, enhancing gastric motility and promoting nutrient absorption [2]. Among these ingredients, licorice is widely used in Kampo formulations for its harmonizing and anti‐inflammatory properties. Glycyrrhizin, the principal active compound of licorice, is metabolized to glycyrrhetinic acid. The metabolites of glycyrrhetinic acid (e.g., 18β‐glycyrrhetyl‐3‐O‐sulfate) inhibit 11β‐hydroxysteroid dehydrogenase type 2 (11β‐HSD2), resulting in licorice‐induced pseudoaldosteronism [3]. This inhibition disrupts the conversion of active cortisol to its inactive form, cortisone, allowing excessive activation of the mineralocorticoid receptor. As a result, sodium retention increases, while renal excretion of potassium and hydrogen ions is enhanced, which leads to hypertension, hypokalemia and metabolic alkalosis. TJ‐43 contains 1370 μg of glycyrrhizin per gram; the estimated glycyrrhizin intake in this case was 0.21 mg/kg/day [2]. Hypoalbuminemia (serum albumin 1.9 g/dL at onset) may increase the unbound fraction of glycyrrhetinic acid–related metabolites and enhance their distribution into principal cells where 11β‐HSD2 is located, thereby increasing susceptibility to pseudoaldosteronism [3]. In addition, the major hepatic metabolism of glycyrrhetinic acid involves sulpho‐conjugation at C‐3 by SULT2A1, which is lower in neonates than in adults; glucuronidation is also immature in neonates, particularly in preterm infants [3, 4, 5]. These developmental factors may cause delayed clearance and prolonged symptoms.

In this case, renin activity and aldosterone levels were not measured. The absence of other potassium‐wasting drugs and improvement after discontinuation of rikkunshito strongly suggest licorice‐induced pseudoaldosteronism. Although rikkunshito may benefit preterm infants by improving gastrointestinal motility, clinicians should recognize that the licorice component can potentially cause serious adverse effects. Evidence for optimal dosing and duration in neonates remains limited, warranting further investigation.

## Author Contributions

T.N. collected the clinical data and prepared the initial manuscript and figures. T.N., N.H. and S.N. participated in the clinical diagnosis and management of the patient. T.Y. and G.Y. critically revised the manuscript. All authors have read and approved the final version of the manuscript.

## Ethics Statement

This case report was approved by the Institutional Review Board of Tokyo Medical University (Approval No. T2024‐0049).

## Consent

Written consent for publication was obtained from the patient's parents.

## Conflicts of Interest

The authors declare no conflicts of interest.

## Data Availability

Research data are not shared.
